# Evaluation of Endotracheal Tube Cuff Pressure and the Use of Three Cuff Inflation Syringe Devices in Dogs

**DOI:** 10.3389/fvets.2020.00039

**Published:** 2020-02-06

**Authors:** Wan-Chu Hung, Jeff C. Ko, Ann B. Weil, Hsin-Yi Weng

**Affiliations:** ^1^Department of Veterinary Clinical Sciences, College of Veterinary Medicine, Purdue University, West Lafayette, IN, United States; ^2^Department of Comparative Pathobiology, College of Veterinary Medicine, Purdue University, West Lafayette, IN, United States

**Keywords:** endotracheal tube cuff pressure, endotracheal tube cuff inflation device, endotracheal tube cuff pressure monitoring, endotracheal tube cuff pressure measurement, dog

## Abstract

Over-inflation of an endotracheal tube (ETT) cuff may lead to tracheal mucosal irritation, tracheal wall ischemia or necrosis, whereas under-inflation increases the risk of pulmonary aspiration as well as leaking anesthetic gas and polluting the environment. The objectives of this two-phase study were to (1) identify the incidence of improper ETT cuff inflation (both over- and under-inflation) using the minimum occlusive volume (MOV) technique coupled with a regular injectable syringe in the anesthetized dogs, and (2) evaluate the performance of two commercially available inflation syringe devices (Tru-Cuff and AG Cuffill®) with the regular injectable syringe in inflating the ETT cuff to a recommended safe cuff pressure range (20–30 cmH_2_O). Dogs undergoing general anesthesia at Purdue Veterinary Medicine Teaching Hospital were included. The ETT cuff pressure was assessed with an aneroid manometer after the syringe inflation. The results of the first objective showed that a total of 80 dogs enrolled and that 50 of these 80 dogs required ETT cuff inflation. Among the 50 dogs, only 14% had proper ETT cuff inflation; 76% of the ETT cuffs were over-inflated and 10% were under-inflated. Ninety dogs were enrolled for the second objective study and they were randomly and equally assigned to the three syringe device treatment groups. The results showed that 80% of the ETT cuffs were over-inflated in the regular injectable syringe treatment group, whereas only 6.7% and 3.3% ETT cuffs were over-inflated in the Tru-Cuff and AG Cuffill® syringe treatment groups, respectively. The AG Cuffill® syringe treatment group had a significantly (*p* < 0.05) higher percentage of properly inflated ETT cuffs (86.7%) compared to the other two groups (regular injectable syringe [3.3%]; Tru-Cuff syringe [50%]. We concluded that there was a high incidence of improper ETT cuff inflation when using MOV technique coupled with a regular injectable syringe. The use of an AG Cuffill® syringe significantly reduced improper ETT cuff inflation.

## Introduction

Endotracheal intubation is routinely performed during general anesthesia. When the cuff on the endotracheal tube (ETT) is inflated, it is crucial to maintain a proper cuff pressure because both excessively high (over-inflation) or low (under-inflation) cuff pressure can lead to serious adverse events. Endotracheal intubation in cats undergoing minor procedures has been reported to be associated with increased odds of anesthetic-related death (1). The adverse events caused by over-inflation of the ETT cuff, including tracheal mucosal irritation, tracheal necrosis, tracheal stenosis, and tracheal rupture, have been reported in dogs, horses, and cats (2–5). Under-inflation of the ETT cuff has been found to increase the risk of pulmonary aspiration in humans (6).

The ideal ETT cuff pressure should be high enough to seal the trachea but not impede the tracheal mucosal blood flow. The tracheal capillary perfusion pressure in humans ranges from 22 to 32 mmHg (30–43.5 cmH_2_O) and in the rabbit ranges from 14 to 28 mmHg (19–38 cmH_2_O) (7, 8). High ETT cuff pressure has been found to impede tracheal mucosal blood flow in both humans and dogs (8, 9). Currently, there is no consistent recommendation for the standard ETT cuff pressure in veterinary medicine (10–12). Based on the majority of human literature, ETT cuff pressure between 20 and 30 cmH_2_O is considered to be the standard (safe) ETT cuff pressure range (13–15).

**Figure 1:**
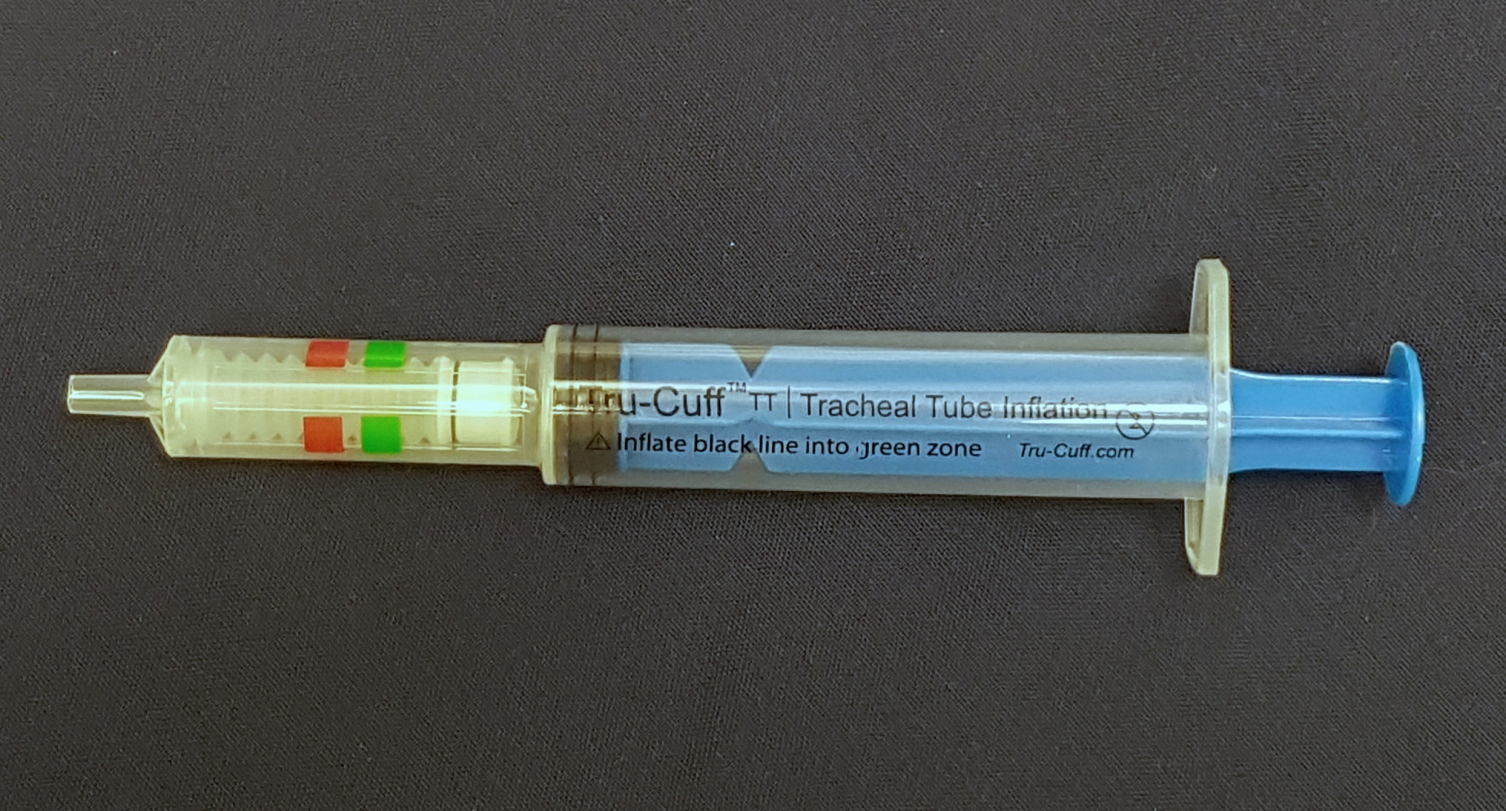
The Tru-Cuff^TM^ syringe has green (18–26 cmH_2_O) and red color (40–60 cmH_2_O) zones indicating different ETT cuff pressure ranges.

**Figure 2:**
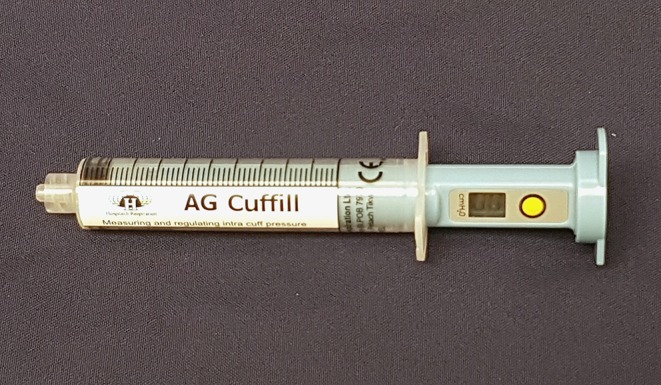
The AG Cuffill syringe has a pressure sensor in the device which can detect and display the ETT cuff pressure value on the screen of the plunger. The yellow button allows the reset of the pressure value.

In veterinary medicine, ETT cuff inflation is usually performed with a subjective estimation of the cuff pressure. One of the commonly used ETT cuff inflation techniques is palpation of the pilot balloon. Another commonly used technique is the minimum occlusive volume (MOV) technique. It is performed by inflating the ETT cuff with a regular injectable syringe until there is no audible air leakage noise when applying a positive airway pressure of 15–30 cmH_2_O in a breathing circuit (12, 16). However, in the recent studies in dogs and cats, it was found that the MOV technique was inaccurate in inflating the ETT cuff to the recommended cuff pressure and tended to over-inflate the ETT cuff (17, 18). Currently, there are several commercial syringe devices specifically designed for ETT cuff inflation with pressure values shown on the syringe device. The Tru-Cuff™ syringe is a cuff inflation syringe device with red and green color zones on the syringe barrel indicating safe and dangerous cuff pressure ranges, respectively (Figure 1). The efficacy of the Tru-Cuff™ syringe has been validated in a human study and it was recommended as an affordable and reliable syringe device for ETT cuff inflation for pediatric patients (19, 20). The AG Cuffill syringe is manufactured with a pressure sensor in the device and is able to display the ETT cuff pressure on the screen in the syringe plunger during cuff inflation (Figure 2). Both syringe devices are FDA approved for use in humans but haven't been evaluated in veterinary medicine.

In humans, some factors can affect the ETT cuff pressure during anesthesia, including changes in the patient's body position, the use of nitrous oxide as an anesthetic adjuvant, and prolonged mechanical ventilation of the intubated patient (21–23). In veterinary medicine, the change of ETT cuff pressure during anesthesia has only been investigated in one study in dogs, and it was found that the ETT cuff pressure decreased over time when anesthetized dogs were breathing spontaneously (24).

Review of the literature in veterinary medicine reveals that there is no standardized recommendation or guideline of routine monitoring ETT cuff pressure in anesthetized patients. The incidence of over- or under-inflation of the ETT cuff with various inflation methods in the anesthetized dogs remains unknown. The objectives of this two-phase prospective study were (1) to determine the incidence of over- and under-inflation of ETT cuff in dogs undergoing general anesthesia at a veterinary teaching hospital using MOV technique coupled with a regular injectable syringe, and (2) to evaluate the performance of three different commercially available syringe devices (regular injectable syringe, Tru-Cuff™ syringe, and AG Cuffill syringe) in inflating the ETT cuff to a recommended safe cuff pressure range (20–30 cmH_2_O) used in human and veterinary literatures (see M&M). Besides these two objectives, we also evaluated the ETT cuff pressure changes over time under two different ventilation modes (spontaneous vs. mechanical ventilation) and after a body position change. In addition, we investigated a group of dogs that did not require initial ETT cuff inflation after endotracheal intubation for 30 min to see if cuff inflation was required during this time period. We hypothesized that there was a high incidence of improper ETT cuff inflation (both over-inflation and under-inflation) in the anesthetized dogs associated with MOV technique coupled with a regular injectable syringe, and the use of one of the commercial cuff inflation syringe devices (Tru-Cuff™ or AG Cuffill syringe) would help reduce the incidence of improper ETT cuff inflation in the anesthetized dogs compared to using a regular injectable syringe.

## Materials and Methods

### Animals and Anesthetic Induction Protocols

This study was approved by the Purdue Animal Care and Use Committee (PACUC) (Protocol number: 1804001729). A total of 209 dogs that admitted to Purdue Veterinary Medicine Teaching Hospital (PVMTH) between June and December 2018 and received general anesthesia were enrolled in this study. Only dogs intubated with SurgiVet® silicone cuffed ETTs without wire enforcement were included. Dogs with upper airway obstruction, those undergoing tracheotomy or thoracotomy, and those with pneumothorax were excluded from this study.

A 9-point body condition scoring system (BCS) were used in this study (25). The dog breeds including American Bulldog, Boston Terrier, Boxer, Chihuahua, English Bulldog, Mastiff, Pomeranian, Shih Tzu, and Staffordshire Bull Terrier were categorized as brachycephalic breeds (26–28) and all other breeds were categorized as non-brachycephalic breeds.

The dogs in this study were premedicated with various sedatives including dexmedetomidine, midazolam, acepromazine, and opioids based on the choice of the anesthesiologists on duty. For intravenous induction agents used in this study, 113 out of all 209 dogs (combining both Phase I and Phase II, see below), were induced with propofol with midazolam. Sixty-eight dogs were induced with propofol only. Seven dogs were induced with ketamine and midazolam. Five dogs were induced with alfaxalone and midazolam, 3 dogs were induced with alfaxalone along, 6 dogs were induced with etomidate, and 1 dog was induced with Telazol® (Tiletamine/Zolazepam). Six out of all 209 dogs were induced with sevoflurane.

### Endotracheal Tube Selection and Cuff Pressure Measurement

For this study, the endotracheal tube selection was based on three commonly used methods: estimation of the lean body mass of the dog and two previously published methods (12, 29). The first published method is based on the width of the nasal septum of the dog's nose being equal to the outer diameter of the endotracheal tube. The second method selects an endotracheal tube size based on palpation of the outer diameter of the animal's trachea in the mid-neck region. A study that evaluated these two methods found that direct palpation at the mid-neck region of the trachea is more accurate, therefore to compensate for the variation in endotracheal tube selection, several sizes of endotracheal tubes above and below the target size of ETT were made available and the one that was the best fit for the trachea during the intubation was used (29). The ETT cuff pressure was measured and constantly monitored with a commercial ETT cuff pressure manometer (Posey Cufflator™ Endotracheal Tube Inflator and Manometer) following the manufacturer's instruction. The accuracy of the manometer was verified with a mercury sphygmomanometer daily prior to use.

For the cuff inflation and the pressure validation procedure, the assigned syringe device was first connected to the pilot balloon one-way valve port to inflate the cuff, and then the syringe device was detached. Following this, the Posey Cufflator™ was then attached to the same one-way valve and the cuff pressure that registered on the Posey Cufflator™ manometer was recorded. Care was taken by pinching the pilot balloon tubing each time during the disconnection and reconnection between the pilot balloon one-way valve and the Posey Cufflator port to avoid an inadvertent air leakage from the one-way valve and a subsequent reduction in the cuff pressure. The Posey Cufflator™ was left attached for continuous monitoring with the pressure value recorded every 5 min for the next 120 min, or until the anesthetic procedure ended, whichever occurred first.

The “standard” or “proper” ETT cuff pressure used in this study was based on published literature in both human and veterinary medicine (13, 18). An ETT cuff pressure between 20 and 30 cmH_2_O was defined as normal inflation. An ETT cuff pressure higher than 30 cmH_2_O was defined as over-inflation, whereas a cuff pressure below 20 cmH_2_O was defined as under-inflation. Both over-inflation and under-inflation were considered as improper ETT cuff inflation in this study. Because the maximum pressure that Posey Cufflator™ could measure is 120 cmH_2_O, an ETT cuff pressure higher than this threshold was recorded as >120 cmH_2_O.

### Phase-One Study–Assessment of the Incidence of Improper Cuff Pressure

A total of 80 dogs were enrolled in Phase-one from June to August 2018. For each dog, the anesthetic protocol was determined by the board-certified anesthesiologist on duty based on the health condition of the dog. After the dog was anesthetized and orotracheally intubated, the ETT cuff inflation was performed using a common leak checking method followed by the MOV technique. The leak check was first applied to the dog immediately after endotracheal intubation while the dog was in either sternal or lateral recumbency and connected to the anesthetic breathing circuit to determine whether ETT cuff inflation was required for the dog. The leak checking was carried out by closing the pop-off valve (i.e., adjustable pressure limiting valve) of the breathing circuit of an anesthetic machine and manually compressing the reservoir bag to a peak airway pressure of 20 cmH_2_O (registered on the breathing circuit pressure manometer) at the same time. The noise of air leakage was checked by a person listening to the dog's muzzle area. If there was an audible air leakage noise, ETT cuff inflation was carried out by using a regular injectable syringe coupled with the MOV technique.

**Figure 3:**
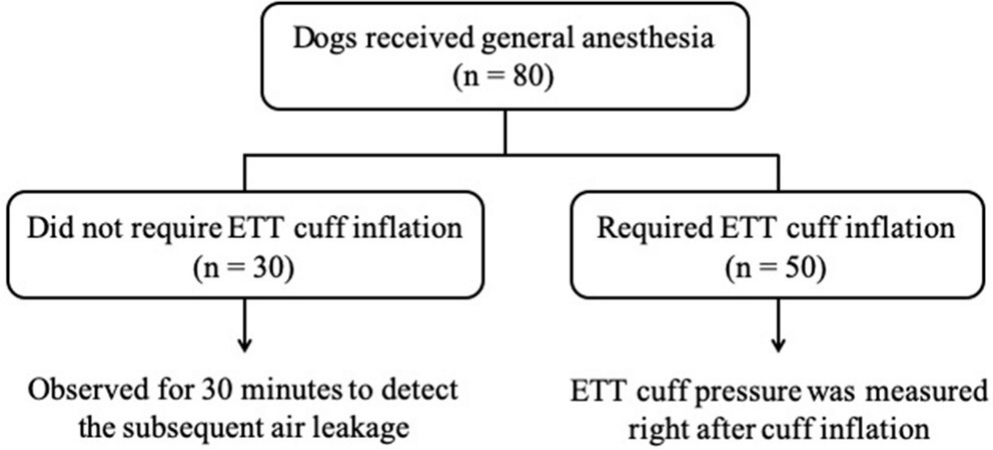
The flow chart of Phase-one study design. The number of dogs in each section is indicated by *n*.

The dogs enrolled in the Phase-one were first divided into two groups based on the result of the leak check: the first group was the dogs that required ETT cuff inflation (*n* = 50) and the other group was the dogs that did not (*n* = 30). For the dogs that required ETT cuff inflation, the ETT cuff pressure was measured right after cuff inflation with the Posey Cufflator™. Dogs that did not require ETT cuff inflation were closely monitored for 30 min and rechecked if cuff inflation was required during this time period. The study design of the Phase-one is illustrated in Figure 3.

### Phase-Two: Comparison of Cuff Pressure Using the Three Syringe Devices and Under Different Ventilation Modes

#### Comparisons of Cuff Pressure Using Three Inflation Syringe Devices

One hundred twenty-nine dogs enrolled in the PVMTH from August to December 2018 were included in this phase. Similar to the Phase-one study, the anesthetic protocol and whether the dog required mechanical or spontaneous ventilation was determined by the board-certified anesthesiologist on duty. After the dog was anesthetized and intubated, a leak check was performed to determine whether the patient required ETT cuff inflation or not (the procedures described in Phase-one study). Dogs that required ETT cuff inflation (*n* = 90) were randomly assigned to one of the three inflation syringe device treatment groups with a balanced number in each group (30 dogs/group). Dogs that did not require ETT cuff inflation (*n* = 39) were excluded from the randomization but the signalments and the anesthetic protocols of these dogs were recorded.

In the first treatment group, the ETT cuff was inflated with a regular injectable syringe (using either a 6-ml or 12-ml syringe) and the MOV technique was applied as described previously in the Phase-one study. In the second and third treatment groups, the ETT cuff was inflated with the Tru-Cuff™ syringe and AG Cuffill syringe, respectively. The manufacturer's instructions were closely followed when using both cuff inflation syringe devices.

**Figure 4:**
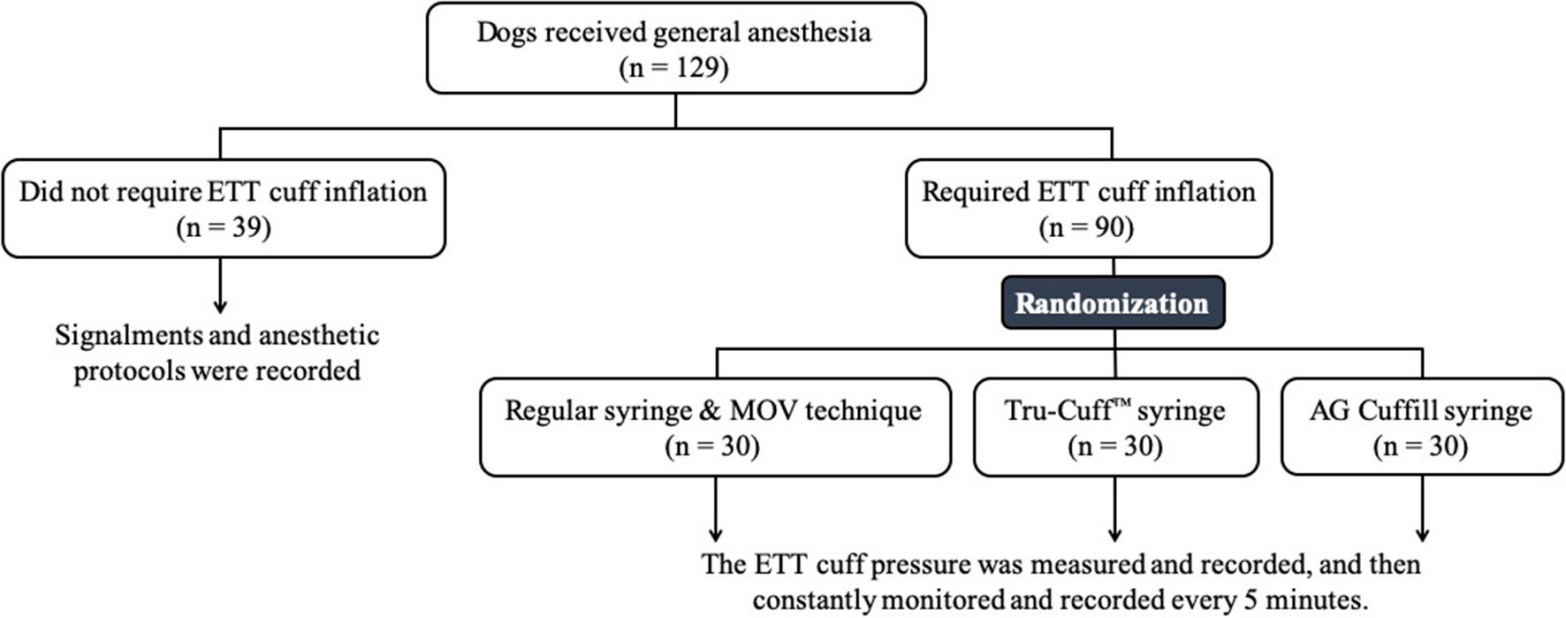
The flow chart of Phase-two study design. The number of dogs in each section is indicated by *n*.

In the second treatment group, the ETT cuff was inflated with the Tru-Cuff™ syringe until the black marker on the plunger reached the top margin of the green zone (safe pressure zone- Figure 1). In the third treatment group, the ETT cuff was inflated with an AG Cuffill syringe until the manometer on the plunger displayed the digital value of 30 cmH_2_O, which indicated the cuff pressure detected by the syringe device (Figure 2). When using the Tru-Cuff syringe, caution was taken to inject air slower than with the regular injectable syringe to allow the bellows to equilibrate with cuff pressure. The person who assessed the ETT cuff pressure was not blinded to the treatment assignment. However, since the ETT cuff pressure was an objective measurement, measurement bias due to non-blinding was not a major concern. The study design in Phase-two is illustrated in Figure 4.

#### ETT Cuff Pressure Changes Associated With Ventilation Modes

In Phase-two, the ETT cuff pressure changes under either mechanical or spontaneous ventilation were investigated. Only dogs under the same ventilation mode for 60 min or longer were included in the analysis. The ETT cuff pressure changes were defined as the cuff pressure difference when measured at the 60th minute and the cuff pressure measured at the beginning when the dog was transferred to the operating room.

#### Cuff Pressure Changes Associated With Body Position Changes

The effect of body position change on the ETT cuff pressure was also evaluated in the Phase-two study. The ETT cuff pressure values before and immediately after a body position change during anesthesia were recorded and compared. The directions of the body position change were categorized as sternal to dorsal, left/right lateral to sternal, sternal to left/right lateral, one side lateral to the other side lateral and dorsal to sternal recumbency.

### Statistical Analysis

Statistical analysis was performed using Stata 15.0 (Stata Corp LLC, College Station, TX, USA) with a statistical significance level set at *p* < 0.05. Prior to the study, a power analysis was performed to determine the sample size required in the Phase-two study to detect a clinically important effect using a chi-square test of independence, with the power of 80% and a significance level of 0.05. Based on the calculation, the total number of 90–135 dogs with 30–45 dogs in each treatment group would yield the desired power. The primary outcome variable in both Phase-one and Phase-two studies was the ETT cuff inflation conditions (over-inflation, normal inflation, under-inflation), expressed as frequency (percentage). The secondary outcome variable in Phase-two was the ETT cuff pressure change (over 60 min under two ventilation types), expressed as mean ± SD. Covariates and other signalments of the dogs were expressed as frequency (percentage) for categorical variables (e.g., sex, breeds, ASA physical status, BCS, procedures performed, drugs, body position, intravenous induction protocols), and as median (range) for continuous variables and ordinal variables (e.g., age, BW, ETT size, BCS). Chi-square tests and Fisher's exact tests were used to assess the association between the usage of different cuff inflation syringe devices and ETT cuff inflation conditions. For the significant test results, logistic regression was then used to perform pairwise comparisons between the treatment groups. Bonferroni adjustments were applied. For pairwise comparisons, two binary variables were created: over-inflation: yes/no and normal inflation: yes/no. Chi-square tests and Fisher's exact tests were also used to compare the covariates that were expressed as categorical variables between groups. Student's *t*-tests were used to compare the ETT cuff pressure changes over 60 min under two ventilation types. For the covariates that were expressed as continuous and ordinal variables between two groups, Student's test and Kolmogorov-Smirnov test were the parametric and non-parametric tests used, respectively. For the covariates that were expressed as the continuous and ordinal variables between three groups, ANOVA and Kruskal–Wallis test was the parametric and non-parametric test used, respectively.

## Results

### Phase-One: Assessment of the Incidence of Improper Cuff Pressure

Of the 50 dogs that required ETT cuff inflation in this phase, 26 (52%) were males and 24 (48%) were females. The median age was 6 years (range, 3 months to 17 years). The median body weight (BW) was 22.95 kg (50.60 lb; range, 1.64–50.3 kg [3.62–110.89 lb]) and the median BCS was 5 (range, 3–8). The breeds of the dogs enrolled are listed in Table 1. The median size of the ETT used was 11 mm internal diameter (ID; range, 4–14 mm). Due to the size of the enrolled dogs, sizes 11 and 12 mm ID were most commonly used (*n* = 20; 40%).

**Table 1:** The breeds and the number of dogs in the Phase-one study.

**Breed**	**No. of cases**
Mixed breed	10
German Shepherd	5
Labrador Retriever	5
Golden Retriever	3
Yorkshire Terrier	3
Greyhound	2
Airedale Terrier	1
American Pit Bull Terrier	1
Basset Hound	1
Beagle	1
Belgian Malinois	1
Boston Terrier	1
Boxer	1
Cavalier King Charlies Spaniel	1
Chesapeake Bay Retriever	1
Dachshund	1
English Bulldog	1
Goldendoodle	1
Great Dane	1
Newfoundland	1
Papillon	1
Rat Terrier	1
Shih Tzu	1
Siberian Husky	1
Soft-coated Wheaten Terrier	1
Toy Poodle	1
West Highland Terrier	1
Whippet	1

*There was a total of 28 breeds enrolled in Phase-one*.

The distribution of the ETT cuff inflation conditions is summarized in Table 2. Of the 50 dogs studied, 14% [*n* = 7; 95% confidence interval (CI), 6.64–27.13%] of the ETT cuffs were normally inflated based on our definition, 76% (*n* = 38; 95% CI, 61.79–86.11%) were over-inflated and 10% (*n* = 5; 95% CI, 4.09–22.44%) were under-inflated. Collectively, the total percentage of improper ETT cuff inflation conditions was 86%. There was no significant difference in the signalments, intravenous induction protocols, and ETT size among the three ETT cuff inflation conditions.

**Table 2:** The distribution of ETT cuff inflation conditions of the 50 dogs in Phase-one study.

**ETT cuff inflation condition**	**No. of dogs**	**Percentage (95% CI)**
Normal inflation	7	14 (6.64–27.13)
Over-inflation	38	76 (61.79–86.11)
Under-inflation	5	10 (4.09–22.44)

### Phase-Two: Comparisons of Three Syringe Devices in Cuff Inflation

Of the 90 treatment group dogs in this phase, 51 (56.7%) were males and 39 (43.3%) were females. The median age of the dogs was 6 years (range, 3 months to 16 years 5 months). The median BW was 23.95 kg (52.8 lb; range, 2.5–62.5 kg [5.51–137.79 lb]) and the median BCS was 5 (range, 3–8). The breeds of the dogs enrolled are listed in Table 3. The median ETT size used in this phase was 11 mm ID (range, 5–16 mm). Similar to Phase-one, the 11 mm and 12 mm ID were most commonly used (*n* = 44; 48.9%).

**Table 3:** The number and breeds of dogs in Phase-two study.

**Breed**	**No. of cases**
Mixed breed	26
German Shepherd	6
Greyhound	5
Dachshund	4
Great Dane	3
Labrador Retriever	3
American Cocker Spaniel	2
American Pit Bull Terrier	2
Beagle	2
Border Collie	2
Chihuahua	2
English Bulldog	2
Golden Retriever	2
Rottweiler	2
Siberian Husky	2
American Bulldog	1
American Eskimo	1
Australia Heeler	1
Australian Shepherd	1
Bichon Frise	1
Bluetick Hound	1
Borzoi	1
Cairn Terrier	1
Cardigan Welsh Corgi	1
Catahoula Leopard	1
Dalmatian	1
English Cocker Spaniel	1
English Shepherd	1
French Bulldog	1
Havanese Terrier	1
Irish Setter	1
Jack Russell Terrier	1
Leonberger	1
Maltese	1
Miniature Poodle	1
Pomeranian	1
Portuguese Water Dog	1
Staffordshire Bull Terrier	1
Standard Poodle	1
Weimaraner	1

*There was a total of 40 breeds enrolled in Phase-two*.

There was no significant difference in signalments and the ETT size among the three syringe device treatment groups. The distribution of ETT cuff inflation conditions by the three syringe device treatment groups is summarized in Table 4. The percentage of over-inflation was significantly higher in the regular syringe treatment group (80%) compared to the other two treatment groups (Tru-Cuff™ syringe treatment group [6.7%] and AG Cuffill syringe treatment group [3.3%]; both *p* < 0.001). The percentage of normal inflation was significantly higher in the AG Cuffill syringe treatment group (86.7%) compared to the other two treatment groups (Tru-Cuff™ syringe treatment group [50%; *p* = 0.012] and regular syringe treatment group [3.3%; *p* < 0.001]). The Tru-Cuff™ syringe treatment group also had a significantly higher percentage of dogs having normally inflated cuffs (50%) than in the regular syringe group (3.3%; *p* = 0.006).

**Table 4:** The distribution of ETT cuff inflation conditions after the regular injectable syringe, Tru-Cuff™ and AG Cuffill syringe treatments.

**Treatment group (30 dogs in each group)**	**No. of dogs**	**Percentage (95% CI)**
**ETT cuff inflation condition**		
Regular syringe		
Normal inflation	1	3.3 (0.4–22.2)
Over-inflation	24^a^^,^^b^	80 (60.8–91.2)
Under-inflation	5	16.7 (6.7–35.7)
Tru-Cuff™ syringe		
Normal inflation	15^c^	50 (31.9–68.1)
Over-inflation	2	6.7 (1.5–24.7)
Under-inflation	13	43.3 (26.2–62.2)
AG Cuffill syringe		
Normal inflation	26^d^^,^^e^	86.7 (68.0–95.2)
Over-inflation	1	3.3 (0.4–22.2)
Under-inflation	3	10 (2.0–28.3)

*Chi-square and Fisher's exact tests were used to compare the ETT cuff conditions between the three syringe device treatment groups. Logistic regression was then used to perform pairwise comparisons between the treatment groups and Bonferroni adjustments were applied*.

a *Significant difference (p < 0.001) between the regular syringe and Tru-Cuff™ syringe treatment groups*.

b *Significant difference (p < 0.001) between the regular syringe and AG Cuffill syringe treatment groups*.

c *Significant difference (p = 0.006) between Tru-Cuff™ syringe and regular syringe treatment groups*.

d *Significant difference (p < 0.001) between AG Cuffill syringe and regular syringe treatment groups*.

e *Significant difference (p = 0.012) between AG Cuffill syringe and Tru-Cuff™ syringe treatment groups*.

The cuff pressure of the 30 dogs inflated with AG Cuffill digital pressure readout set to 30 cm H_2_O was also compared with the reading obtained with Posey Cufflator™ manometer. It was found that the cuff pressure values were between 2 cmH_2_O higher to 6 cmH_2_O lower than the value 30 cm of H_2_O registered with Posey Cufflator manometer.

### Comparisons of Cuff Pressure Changes Under Two Ventilation Modes

**Figure 5:**
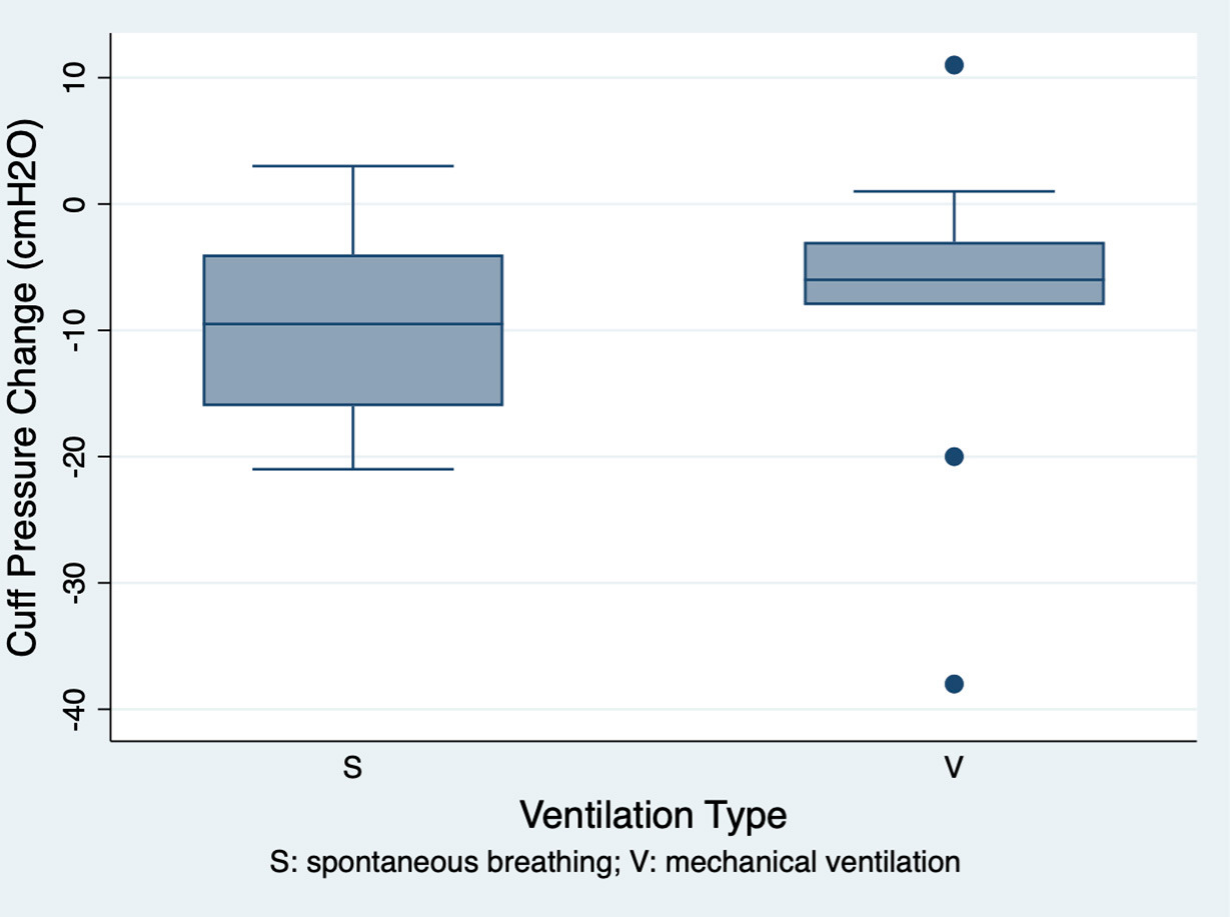
The ETT cuff pressure changes over 60 min in the spontaneous breathing (mean ± SD, −9.6 ± 8.2 cmH_2_O; *n* = 8) and mechanical ventilation (mean ± SD, −8.4 ± 13.8 cmH_2_O; *n* = 9) groups. There was no significant difference in ETT cuff pressure changes over 60 min between the two groups (*p* = 0.836).

Seventeen of the 90 treatment group dogs in Phase-two were selected for evaluating their ETT cuff pressure changes over 60 min. Mechanical ventilation was applied to nine dogs and the other eight dogs were allowed to breathe spontaneously with occasional intermittent manual assisted ventilation. No significant difference was noted in signalments and the ETT size between the two ventilation mode groups. The ETT cuff pressures decreased over 60 min regardless of the mode of ventilation. There was no significant difference in the decreased ETT cuff pressure values between the dogs breathing spontaneously (−9.6 ± 8.2 cmH_2_O) and those with mechanical ventilation (−8.4 ± 13.8 cmH_2_O; *p* = 0.836) (Figure 5).

### ETT Cuff Pressure Changes After Changing Body Position

The changes in body position were observed in 18 of the 90 treatment group dogs in Phase-two. The median ETT cuff pressure change after position change was 0 cmH_2_O (range, −11 to 3 cmH_2_O). The changes in body position and the subsequent ETT cuff pressure changes are summarized in Table 5. One dog developed a subsequent air leakage (detected by the presence of audible air leaking noise during ventilation) after position changed from sternal to dorsal recumbency.

**Table 5:** The ETT cuff pressure changes after the body position changes in 18 dogs in Phase-two.

**The direction of the body position change**	**No. of dogs**	**ETT cuff pressure change before and immediately after body position change (cmH_**2**_O)**
Sternal to dorsal	8	2, 2, 2, 0, 0, 0, −4, −11
Left/right lateral to sternal	4	3, −2, −4, −8
Sternal to left/right lateral	3	2, 0, −4
One side lateral to the other side	2	1, 0
Dorsal to sternal	1	0

### Investigation of Dogs That Did Not Require Cuff Inflation

Of the total 209 dogs enrolled in this study (both Phase-one and Phase-two), 69 (33%; 95% CI, 26.68–39.84%) of them did not require the initial ETT cuff inflation based on the result of the SOP leak check. The proportion of brachycephalic breeds was significantly higher in dogs that did not require ETT cuff inflation (17/69; 24.6%) than in dogs that required ETT cuff inflation (12/140; 8.6%; *p* = 0.002). Of these 69 dogs, 30 of them were in the Phase-one and were closely monitored for 30 min after endotracheal intubation. Subsequent air leakage was noted in six dogs and the median time from the first leak check immediately after endotracheal intubation to the presence of an air leakage was 7.5 min (range, 4–10 min).

## Discussion

The results of this study support our hypothesis that the incidence of improper ETT cuff inflation using the MOV technique is high (86%, Table 2) in anesthetized dogs. It is important to note that using this technique, most of these ETT cuffs were over-inflated. This result is in agreement with other studies in dogs (16) and cats (17). The unique finding of our study, however, was that the use of the commercial ETT cuff inflation syringes equipped with pressure indication devices significantly reduced the incidence of improper ETT cuff inflation when compared with the use of regular injectable syringes coupled with MOV technique. To our knowledge, this is the first documentation comparing the regular injectable syringe with the specific designed ETT cuff inflation syringe devices for evaluating ETT cuff pressure in dogs.

In clinical practice, the ETT cuff inflation in veterinary anesthesia is usually achieved with either palpation of the pilot balloon or using the MOV technique as a subjective estimation of the cuff pressure. In a human anesthesia study, it was demonstrated that 33/113 (29%) of the ETT cuffs were overinflated and pilot balloon palpation was reported as the most common method to determine appropriate ETT cuff pressure (30). The ETT cuff inflation with the MOV technique has been investigated in dogs and cats. In a prospective clinical study in dogs, Briganti et al. found that the percentage of ETT cuff pressures exceeding the normal cuff pressure range (defined as 19–24 mmHg in the study) while using the MOV technique was 65.5% for silicone tubes and 37% for polyvinyl chloride tubes (17). While our study results are in agreement with that study, the frequency of over-inflation of the ETT cuff in our study (76%) was higher than that reported in Briganti's study (17). This discrepancy is likely due to the different definitions of the normal ETT cuff pressure (19–24 mmHg [25.8–32.6 cmH_2_O] vs. 20–30 cmH_2_O) used for each study. Nevertheless, the incidence of over-inflation of the ETT cuff pressure is high in both studies in dogs. In another clinical study in cats, the reported mean ETT cuff pressure after cuff inflation with MOV technique was 61 cmH_2_O, which greatly exceeded the recommended safe cuff pressure range (20–30 cmH_2_O) (18). Although the MOV technique is widely used clinically, the use of only one ventilation pressure of 20 cm H_2_O for dogs of various weights can lead to the ETT cuff pressure being higher than needed on small dogs and not high enough on large/obese dogs. Furthermore, leak detection with the MOV technique is based on listening to a leak sound which may not be as accurate especially if in the presence of a noisy background. A suggestion has been made to use a stethoscope and listen to the ventral neck region near the laryx of the anesthetized dogs while applying the MOV leak check to audibly improve the ability to detect a leak. Collectively, our study emphasizes that the MOV technique coupled with a regular injectable syringe is an unreliable method for ETT cuff inflation and the use of a direct ETT cuff pressure measurement is highly recommended to avoid improper ETT cuff pressure.

Our results demonstrated that both the Tru-Cuff™ and AG Cuffill syringes can be used to effectively reduce the frequency of over-inflation of the ETT cuff in dogs. When all factors are considered, the AG Cuffill outperformed the Tru-Cuff™ and regular injectable syringes by inflating the ETT cuff pressure more frequently within the defined safe range without causing over- or under-inflation (Table 4). This is likely due to the AG Cuffill syringe is equipped with a more precise digital pressure readout device (Figure 2) than the Tru-Cuff™ syringe, which provides only a color-coded zone for a defined pressure range, or the regular injectable syringe, which provides no ETT pressure indication at all. The cuff pressure value differences based on the AG Cuffill digital readout and the actual pressure registered on the Posey Cufflator manometer were between +2 and −6 cmH_2_O, indicating that the pressure reading between these two devices was relatively close in this clinical setting.

Although the Tru-Cuff™ syringe was able to inflate the ETT cuff pressure within the defined safe pressure range in 50% of the anesthetized dogs, there were 43.3% of the dogs with their ETT cuffs under-inflated. This frequency was higher than that of using a regular injectable syringe (16.7%). In a study evaluating the use of Tru-Cuff™ syringe devices in humans, the authors found that 26% of the pressure measurements were actually less than the Tru-Cuff™ manufacturer reported normal pressure range (19). Most of the discrepancy of these measurements in humans were ≤3 cmH_2_O of the reported lower limit value (19). In accordance with these study results, we conclude that although the use of the Tru-Cuff™ syringe device was able to reduce the frequency of over-inflation, caution should be taken to prevent the under-inflation of ETT cuffs which may potentially lead to pulmonary aspiration if regurgitation occurs.

Our study results also showed that the ETT cuff pressure tended to decrease over 60 min regardless of the ventilation modes. This downward trend of the ETT cuff pressure (breathing spontaneously group: −9.6 ± 8.2 cmH_2_O and mechanical ventilation group: −8.4 ± 13.8 cmH_2_O) is consistent with other reports. In an experimental study in Beagles, Shin et al. found that mean ETT cuff pressure decreased from 25 to 10.9 mmHg in 60 min when dogs were breathing spontaneously (24). In another study in horses, it was noted that the ETT cuff pressure tended to decrease during the first 30 min of anesthesia (4). A similar finding was also documented in mechanically ventilated human patients (23, 31). The possible explanations for such a decrease in ETT cuff pressure include tracheal muscle relaxation and the fatigue of the cuff materials (24).

There was also a possibility of gradual leaks over time resulting from either reattaching the Posey Cufflator™or any pressure measuring devices multiple times to the pilot balloon of the ETT or the pressure measuring device itself causing a gradual reduction in cuff pressure. These slow leaks have been reported in both human and veterinary literature during repeated measure of the cuff pressure (23, 24, 31). The slow leaks of cuff pressure described here likely played a different role initially in our study because the majority of cuffs were overinflated. However, once time elapsed, it could lead to a decrease in cuff pressure with resulting under-inflation. These results emphasize the need for continuous monitoring of the ETT cuff pressure throughout the entire anesthetic procedure. Further studies on the causes of decreased ETT cuff pressure over time are warranted to facilitate the management of ETT cuff pressure.

Besides our main objectives, the current investigation also serves as a pilot study in exploring the factors that may affect the ETT cuff pressure in anesthetized dogs. We found that change in body position did cause a change of the ETT cuff pressure in some dogs, but not all the dogs (Table 5). The ETT cuff pressure changes range from −11 to +3 cmH_2_O with a median pressure of 0 cmH_2_O after various body position changes. However, due to the small number of observations in this study, we couldn't draw a definitive conclusion about which direction of body position change causes the increase or decrease of the ETT cuff pressure.

In human patients, it was found that the ETT cuff pressure increased after changing the body position from supine to prone (32). The explanations for such changes included the compression of the trachea and ETT cuff by the spine, muscles and major vessels, and the increased intra-thoracic pressure in prone position due to the compression of the anterior chest wall and the abdomen (32). Further studies with a larger number of observations may help to elucidate the correlation between the body position changes and the ETT cuff pressure changes in dogs.

In this study, 33% of the total enrolled dogs did not require an initial ETT cuff inflation after endotracheal intubation. This condition has not been reported in any of the previous literature involving dog or cat ETT cuff pressure evaluations. Furthermore, of these dogs that did not require the initial ETT cuff inflation, some of them developed a subsequent air leakage, but all within the first 10 min (ranged from 4 to 10 min). The development of subsequent air leakage may be explained by the relaxation of tracheal muscles induced by the inhalant anesthetic agents (33, 34). We also found the dogs that did not require ETT cuff inflation had a three times higher proportion of brachycephalic breeds than the dogs that required ETT cuff inflation (24.6% vs. 8.6%). Brachycephalic breed dogs are prone to have airway anatomical abnormalities including hypoplastic trachea (27, 35). Dogs with hypoplastic trachea have narrowed tracheal diameters along the entire trachea due to the small tracheal cartilage (c-shaped) with overlapped ends (36, 37). This explained why the ETTs were more likely to fit tightly within the trachea and form a better seal without ETT cuff inflation in the brachycephalic breed dogs than non-brachycephalic breeds.

In this study, we did not specifically recruit dogs to be induced with a variety of different anesthetic protocols since it was not the main goal of this study. However, the statistic result showed that there was no significant impact of intravenous induction protocols (mainly propofol vs. propofol with midazolam) on the ETT cuff values in both Phase I and Phase II studies. The numbers of dogs induced with other intravenous induction agents (alfaxalone, midazolam-ketamine, and tiletamine-zolazepam) are too small to allow further statistical analysis.

The limitations of this study include (1) only one type of the ETT (SurgiVet® silicone cuffed ETT without wire enforcement) was evaluated so the conclusions of this study may not be applied to other types of ETTs (e.g., polyvinyl chloride cuffs or polyurethane), and (2) most of the enrolled dogs were medium to large sizes so the results may be skewed when they were extrapolated to the whole canine population. The silicone cuffed ETT used in this study is unique and deserves a specific discussion. The detailed classifications of human endotracheal tube cuffs have been described based on the cuff shape (spheroidal, cylindrical, tapered), materials (polyvinyl chloride, polyurethane, and silicone), elasticity (high and low) and the cuff type (38). The cuff type, which is solely based on the manufacturer claims, is classified as high-volume low-pressure (HVLP), low-volume high-pressure (LVHP) and low volume-low pressure (38). A HVLP cuff is designed to have a larger surface area in contact with the trachea so when the cuff is inflated it applies a lower pressure against the tracheal wall, and therefore results in a lower incidence of tracheal wall ischemia. On the other hand, an inflated LVHP cuff has a lower volume with a small contact surface area within the trachea, potentially providing it a more effective seal than an HVLP cuff. However, the high pressure may lead to a higher potential of tracheal mucosa ischemia after prolonged use.

Very little information has been described by the manufacturer in the veterinary silicone endotracheal tubes used in this study. In Briganti's study, they used the same type of silicone tube as we did in our study (17). These authors specifically pointed out that the silicone tubes should be considered separately since it does not fall perfectly within the LVHP classification. They also pointed out that several versions of the silicone tubes used in humans (similar to the ones used in this study) are defined as low-volume low- pressure tubes with high elasticity (17, 38, 39). As Briganti et. al. indicated, the main issue for evaluating the inflation of the silicone tube cuffs is the non-linear relationship between the cuff pressure and cuff volume since most of the pressure inside the silicone cuff is associated with overcoming the compliance (or elasticity) of the cuff wall (17). The high elasticity of the silicone cuff, unlike the polyvinyl chloride cuff, requires initial high pressure to expand the silicone sleeve and the pressure does not continue to increase when the cuff diameter reaches the tracheal diameter (40). It was demonstrated that in a silicone cuff of a human ETT, an initial intracuff pressure of 80 cm H_2_O is needed to overcome the elasticity of the silicone cuff. This cuff pressure then provides a calculated tracheal wall seal pressure of approximately 30 cmH_2_O depending on the patient's anatomy (38, 39). It has been clearly illustrated in a human silicone cuff ETT that the pressure-volume curve reaches a plateau at a cuff diameter approximating the tracheal diameter (40). One of the limitations for measuring cuff pressure in the clinical patient is that clinicians assume the ETT cuff pressure is the actual pressure being applied to the trachea. This is likely not the case since there are many other factors involved as we have discussed here. However, inappropriate cuff pressure should always be a concern in clinical anesthesia. The advantage of the high elasticity of a silicone cuff endotracheal tube is that it does not act like the polyvinyl chloride cuff material whereby excess cuff material folds back over itself within the trachea and creates multi-channels that can allow for the micro-aspiration of regurgitant or secretions (38). Regardless of the unique physical property of the silicone cuff, both our study and Briganti's study demonstrated that the MOV technique with a regular syringe consistently resulted in cuff pressures higher than the recommended range (17).

Future studies that include various body sizes and testing on various types of ETTs should provide more representative results of the ETT cuff pressure monitoring in veterinary medicine. Similar studies should also be extended to species, such as cats and horses since various complications associated with improper ETT cuff inflation have also been documented in these species.

## Conclusions

Improper ETT cuff inflation was as high as 86% in the anesthetized dogs when a regular injectable syringe coupled with the MOV technique was used. Both Tru-Cuff™ and AG Cuffill syringes effectively reduced the improper ETT cuff inflation in the same setting. When all three syringe devices were compared, the AG Cuffill syringe had the best performance for inflating ETT cuff to the defined safe pressure range. The ETT cuff pressure decreased over time during anesthesia regardless of the mode of ventilation, therefore continuous monitoring of ETT cuff pressure is strongly recommended. Finally, a total of 33% of the enrolled dogs did not require ETT cuff inflation. Brachycephalic breed dogs were less likely to require ETT cuff inflation when comparing with non-brachycephalic breed dogs. However, a subsequent air leakage can develope within the first 10 min after endotracheal intubation. This finding emphasized the importance of continuous ETT cuff monitoring for anesthetized dogs.

## Data Availability Statement

The datasets generated for this study are available on request to the corresponding author.

## Ethics Statement

The animal study was reviewed and approved by Purdue Animal Care and Use Committee (PACUC). Written informed consent for participation was not obtained from the owners because the procedures involves in this study is part of the routine procedures during anesthesia in the clinical practices (endotracheal tube cuff inflation and leak checking).

## Author's Note

This study is a partial fulfillment of the requirements for the degree of Master of Science of Wan-Chu Hung in the Department of Veterinary Clinical Sciences, College of Veterinary Medicine, Purdue University.

## Author Contributions

W-CH, JK, AW, and H-YW: study design, proposal drafting, interpretation of the results, manuscript writing and revising. W-CH: data collection. H-YW: data analysis.

### Conflict of Interest

The authors declare that the research was conducted in the absence of any commercial or financial relationships that could be construed as a potential conflict of interest.
